# ﻿Review of the wolf-spider genus *Draposa* Kronestedt, 2010 from China (Araneae, Lycosidae)

**DOI:** 10.3897/zookeys.1248.150292

**Published:** 2025-08-08

**Authors:** Zhi-Sheng Zhang, Piao Liu, Shuqiang Li, Lu-Yu Wang

**Affiliations:** 1 Key Laboratory of Eco-environments in Three Gorges Reservoir Region (Ministry of Education), School of Life Sciences, Southwest University, Chongqing 400715, China Southwest University Chongqing China; 2 College of Life Sciences, Anhui Normal University, Wuhu, Anhui 241000, China Anhui Normal University Anhui China

**Keywords:** Distribution, Pardosinae, redescription, synonym, taxonomy

## Abstract

The wolf-spider genus *Draposa* Kronestedt, 2010 in China is reviewed. A new combination is proposed: *Draposaaciculifera* (Chen, Song & Li, 2001), **comb. nov.** (ex. *Pardosa* C L. Koch, 1847). *Pardosashugangensis* Yin, Bao & Peng, 1997 is synonymized with *Draposazhanjiangensis* (Yin, Wang, Peng & Xie, 1995), **syn. nov.** Detailed redescriptions, habitus photographs, illustrations of copulatory organs, and live habitus images are provided for relevant species. *Draposaburasantiensis* (Tikader & Malhotra, 1976) is transferred back to *Pardosa* and figures of *P.burasantiensis* sensu Yin et al. (1997b) from China are provided.

## ﻿Introduction

*Draposa* Kronestedt, 2010 is a small genus with 12 named species distributed from the United Arab Emirates to the Indomalayan Realm (Alderweireldt and Jocqué 2017; WSC 2025). *Draposa* has been well studied in Bangladesh, Bhutan, India, Indonesia, Iran, Maldives, Myanmar, Pakistan, Sri Lanka, and the United Arab Emirates (Kronestedt 2010; Marusik and Omelko 2016; Alderweireldt and Jocqué 2017; Abhijith and Sudhikumar 2023). Currently, only two species of the genus have been recorded in China: *D.burasantiensis* (Tikader & Malhotra, 1976) and *D.zhanjiangensis* (Yin, Wang, Peng & Xie, 1995) (WSC 2025). Here, we review *Draposa* from China based on recently collected specimens, as well as the type specimens of *D.zhanjiangensis*, *Pardosaaciculifera* Chen, Song & Li, 2001, and *P.shugangensis* Yin, Bao & Peng, 1997. We propose that *Pardosaaciculifera* Chen, Song & Li, 2001 should be transferred to *Draposa* and that *Pardosashugangensis* Yin, Bao & Peng, 1997 is a junior synonym of *Draposazhanjiangensis* (Yin, Wang, Peng & Xie, 1995).

## ﻿Materials and methods

All specimens are preserved in 75% ethanol and were examined, illustrated, photographed, and measured using a Leica M205A stereomicroscope equipped with a drawing tube, a Leica DFC450 Camera, and LAS v. 4.6 software. Male palps and epigynes were examined and illustrated after dissection. Epigynes were cleared by immersing them in a pancreatin solution (Álvarez-Padilla and Hormiga 2007). Eye sizes were measured as the maximum dorsal diameter. Leg measurements are shown as: total length (femur, patella and tibia, metatarsus, tarsus). All measurements are in millimetres. Specimens examined here are deposited in the Collection of Spiders, School of Life Sciences, Southwest University, Chongqing, China (**SWUC**); the Institute of Zoology, Chinese Academy of Sciences (**IZCAS**); and the Hunan Normal University (**HNU**).

Terminology follows Kronestedt (2010) and Zhang et al. (2025). Abbreviations used in the text: **ALE**, anterior lateral eye; **AME**, anterior median eye; **MOA**, median ocular area; **PLE**, posterior lateral eye; **PME**, posterior median eye.

## ﻿Taxonomy

### ﻿Family Lycosidae Sundevall, 1833 (狼蛛科)

#### ﻿Genus Draposa

Taxon classificationAnimaliaAraneaeLycosidae

Kronestedt, 2010

F5420D6A-ACF4-54F5-8553-3BB92A76B388

Draposa Kronestedt, 2010: 33.

##### Type species.

*Lycosanicobarica* Thorell, 1891.

##### Diagnosis.

Males of *Draposa* differ from other Pardosinae by the presence of a subpaleal sclerite with two processes (AP and PP, Figs 2B, 3C, E, 5B, 6F, 7C, E) partly hidden by the palea, as well as by the median apophysis (= tegular apophysis in Kronestedt 2010) being transverse with wide basal part carrying variously shaped projections and narrow distal part carrying small subapical protrusion before evenly curved tip; females differ by the epigynal cavity being only partly divided by a tongue-shaped septum.

##### Description.

See Kronestedt (2010).

##### Composition and distribution.

Twelve species are known from the Arabian Peninsula to Indomalayan Region.

#### ﻿ Draposa aciculifera

Taxon classificationAnimaliaAraneaeLycosidae

(Chen, Song & Li, 2001) comb. nov.

8CF97FB7-5E57-577B-8924-526D78CEBF0F

**Figure 1.**
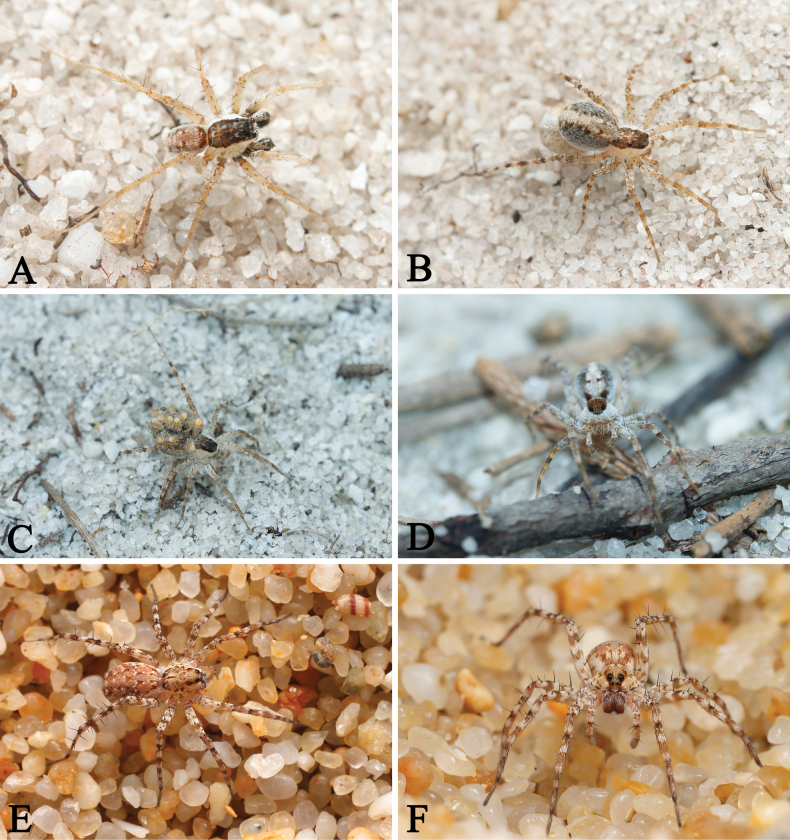
Living *Draposa* spp. A–D. *D.aciculifera* (Chen, Song & Li, 2001) (A. Male, B–D. Female); E, F. *D.zhanjiangensis* (Yin, Wang, Peng & Xie, 1995) (female). Photographed by Qian-Le Lu.

**Figure 2.**
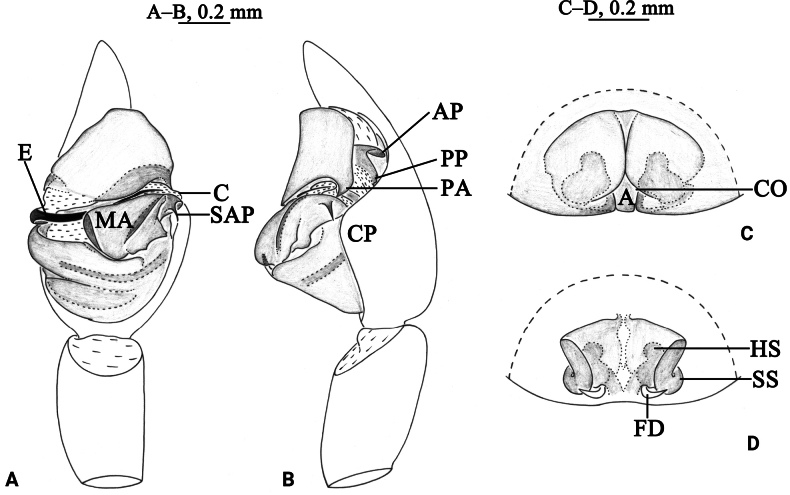
*Draposaaciculifera* (Chen, Song & Li, 2001) male (A, B) and female (C, D). A. Left male palp, ventral view; B. Same, retrolateral view; C. Epigyne, ventral view; D. Same, dorsal view. Abbreviations: A = atrium; AP = anterior subpaleal process; C = conductor; CO = copulatory opening; CP = cymbium protruding; E = embolus; FD = fertilization duct; HS = head of spermathecae; MA = median apophysis; PA = paleal apophysis; PP = posterior subpaleal process; SAP = subapical protrusion on median apophysis; SS = stalk of spermathecae.

**Figure 3.**
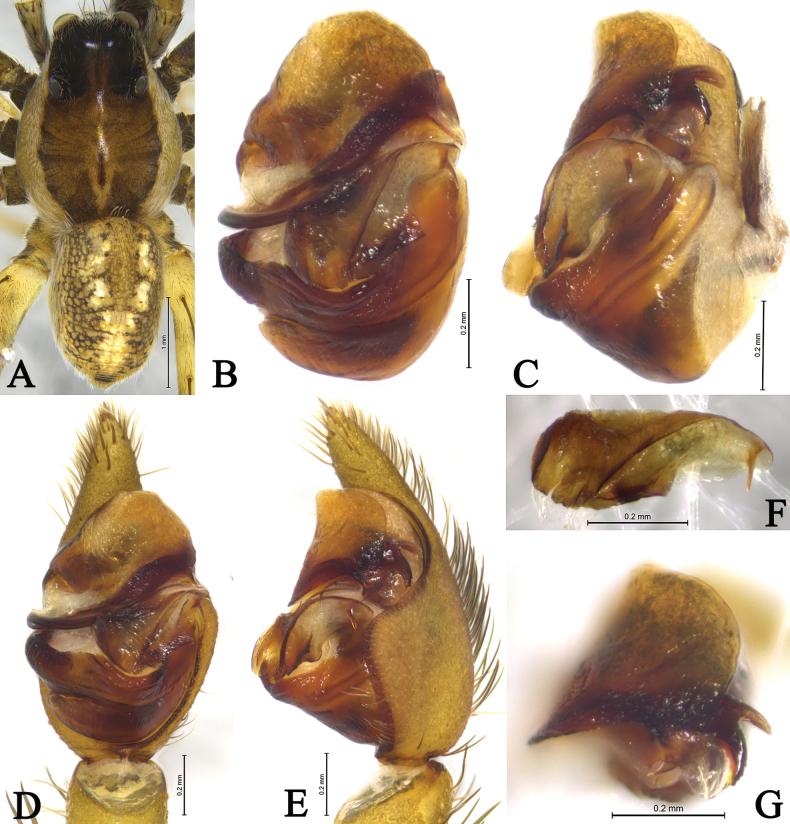
*Draposaaciculifera* (Chen, Song & Li, 2001) male from Kaiyuan City, Yunnan. A. Male habitus, dorsal view; B. Left bulb, ventral view; C. Same, retrolateral view; D. Left male palp, ventral view; E. Same, retrolateral view; F. Median apophysis, ventral view; G. Terminal part in obliquely retrolateral.

**Figure 4.**
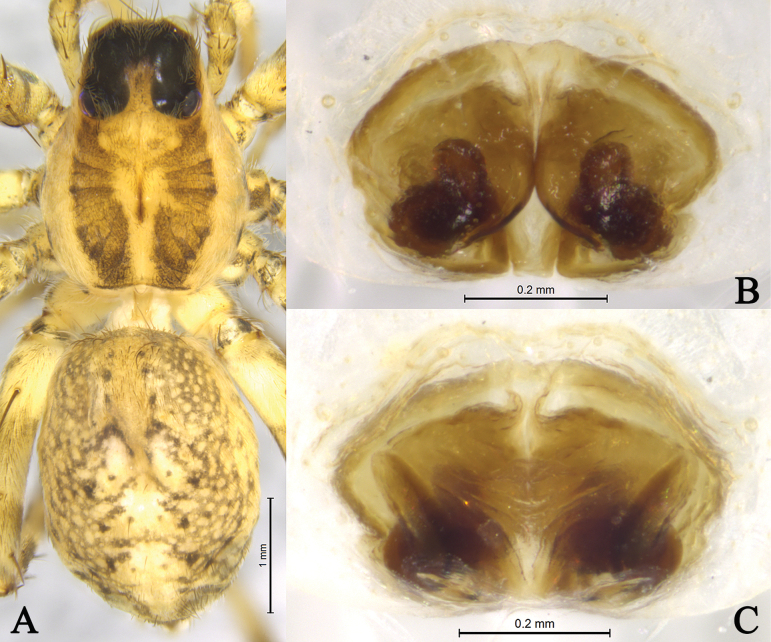
*Draposaaciculifera* (Chen, Song & Li, 2001) female from Kaiyuan City, Yunnan, China. A. Female habitus, dorsal view; B. Epigyne, ventral view; C. Same, dorsal view.

Figs 1A–D , 2 , 3 , 4 , 10

Pardosa aciculifera Chen, Song & Li, 2001: 476, figs 1–7 (♂♀); Wang et al. 2021: 48, fig. 42A–H (♂♀).

##### Material examined.

**China**: • 1♂ 2♀, ***Hainan*** Prov., Jianfengling, 18.7°N, 108.8°E, 12.07.1990 (holotype and 2 paratypes, IZCAS-Ar-9487, IZCAS-Ar-9488, and IZCAS-Ar-9489) • 1♀, ***Hainan*** Prov., Qianghai Co., 20.5.1990 (paratype, IZCAS-Ar-9490) • 2♂ 1♀, ***Yunnan*** Prov., Kaiyuan City, 23°40'54"N, 103°20'27"E, elev. 1389 m, 14.06.2017, L.Y. Wang et al. leg. (SWUC, SWUC-LYDA-01–03) • 5♂ 14♀, ***Guangxi*** Prov., Beihai City, Hepu Co., Shankou Mangrove Nature Reserve, Dunzai Vill., 21°31'2"N, 109°45'36"E, 13.05.2024, Q.L. Lu leg. (SWUC, SWUC-LYDA-04–22) • **Thailand**: 6♂ 6♀, ***Chiang Mai***, Amphoe Hot, 18°09'14"N, 98°25'51"E, elev. 782 m, 5.07.2014, Z.S. Zhang et al. leg. (SWUC, SWUC-LYDA-23–34).

##### Comments.

Kronestedt (2010) has already suggested that this species might belong to *Draposa*.

##### Diagnosis.

*Draposaaciculifera* can be distinguished from all congeners by the acicular subapical protrusion (SAP) of the median apophysis (MA) in the male palp (vs triangular) and the absence of epigynal septum (vs present).

##### Description.

**Male** (Fig. 3A) total length 4.14. Carapace 2.41 long, 1.85 wide; opisthosoma 1.89 long, 1.29 wide. Carapace dark brown, with lateral margin with yellow bands, and covered with white setae; small, light, longitudinal stripe around fovea. Cervical groove and radial furrows indistinct. Eye sizes and interdistances: AME 0.11, ALE 0.07, PME 0.32, PLE 0.28; AME–AME 0.10, AME–ALE 0.04, PME–PME 0.37, PME–PLE 0.45. Clypeus height 0.25. Chelicerae brown. Endites and labium brown. Sternum black. Leg measurements: I 8.86 (1.90, 2.41, 1.76, 1.07); II 6.78 (1.74, 2.27, 1.77, 1.00); III 6.81 (1.76, 2.15, 1.94, 0.96); IV 10.02 (2.51, 2.96, 3.14, 1.41). Opisthosoma oval, yellow-brown and covered with numerous black spots dorsally. Heart mark distinct. Venter yellow-brown.

***Palp*** (Figs 2A, B, 3B–G). Tibia yellow-brown, 1.7 times longer than wide. Cymbium yellow-brown, with strong protruding (CP) of retrolateral margin. Median apophysis (MA) moderately long, 2.4 times longer than wide, with lamellar projection in basal half, its subapical protrusion (SAP) acicular, tip of median apophysis semicircle. Paleal apophysis (PA) corniform; anterior subpaleal process narrow (AP), lamellar, with rounded tip, as long as posterior subpaleal process; posterior subpaleal process (PP) wider than long, half sclerotized and half membranous, with serrated edges. Embolus (E) narrow, originating at approximately 9-o’clock position, without membrane, evenly tapering to tip.

**Female** (Fig. 4A) total length 5.27. Carapace 2.41 long, 1.83 wide; opisthosoma 2.62 long, 1.89 wide. Eye sizes and interdistances: AME 0.10, ALE 0.05, PME 0.32, PLE 0.27; AME–AME 0.11, AME–ALE 0.04, PME–PME 0.37, PME–PLE 0.45. Clypeus height 0.22. Leg measurements: I 6.51 (1.85, 2.23, 1.53, 0.90); II 5.38 (1.48, 1.77, 1.21, 0.92); III 6.21 (1.62, 2.00, 1.68, 0.91); IV 9.69 (2.26, 2.87, 3.15, 1.41).

***Epigyne*** (Figs 2C, D, 4B, C). Plate ~1.6 times wider than long. Atrium (A) narrow; septum absent; copulatory openings (CO) crack-shaped, located on both side of the atrium. Spermathecal heads (HS) spherical, visible through cuticle of lateral walls of atrium. Spermathecal stalks (SS) arc-shaped. Fertilization ducts (FD) crescent.

##### Note.

As described above, copulatory organs of this species are completely consistent with this genus except tongue-shaped septum (absent). Therefore, we transfer *Pardosaaciculifera* to *Draposa*.

##### Distribution.

China (Hainan, Guangxi, Yunnan), Thailand (Chiang Mai) (Fig. 10).

#### ﻿ Draposa zhanjiangensis

Taxon classificationAnimaliaAraneaeLycosidae

(Yin, Wang, Peng & Xie, 1995)

572F06BF-1E4F-5DDC-97B3-2C3B96DFD85B

**Figure 5.**
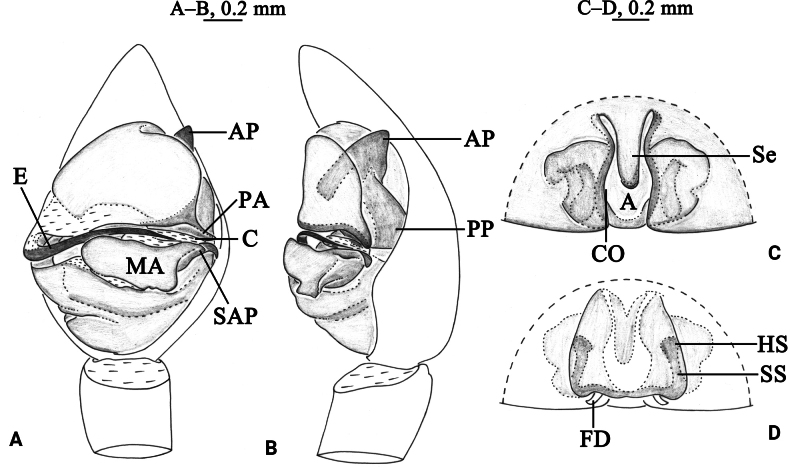
*Draposazhanjiangensis* (Yin, Wang, Peng & Xie, 1995) male allotype (A, B) and female holotype (C, D). A. Left male palp, ventral view; B. Same, retrolateral view; C. Epigyne, ventral view; D. Same, dorsal view. Abbreviations: A = atrium; AP = anterior subpaleal process; C = conductor; CO = copulatory opening; E = embolus; FD = fertilization duct; HS = head of spermathecae; MA = median apophysis; PA = paleal apophysis; PP = posterior subpaleal process; SAP = subapical protrusion on median apophysis; Se = Septum; SS = stalk of spermathecae.

**Figure 6.**
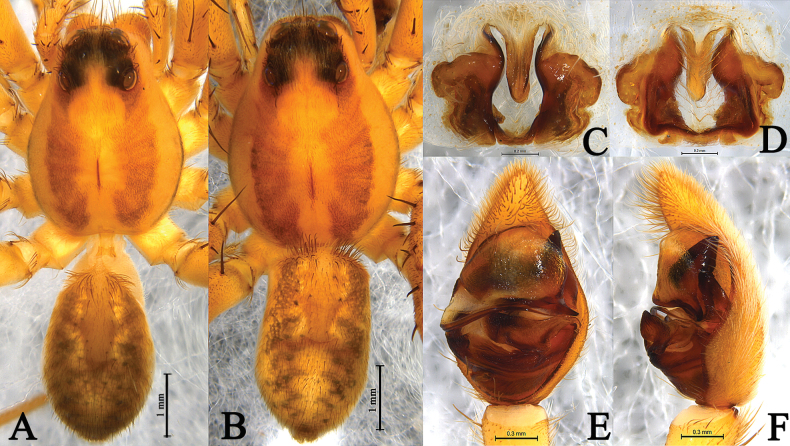
*Draposazhanjiangensis* (Yin, Wang, Peng & Xie, 1995) female holotype (A, C, D) and male allotype (B, E, F). A. Female habitus, dorsal view; B. Male habitus, dorsal view; C. Epigyne, ventral view; D. Vulva, dorsal view. E. Left male palp, ventral view; F. Same, retrolateral view.

**Figure 7.**
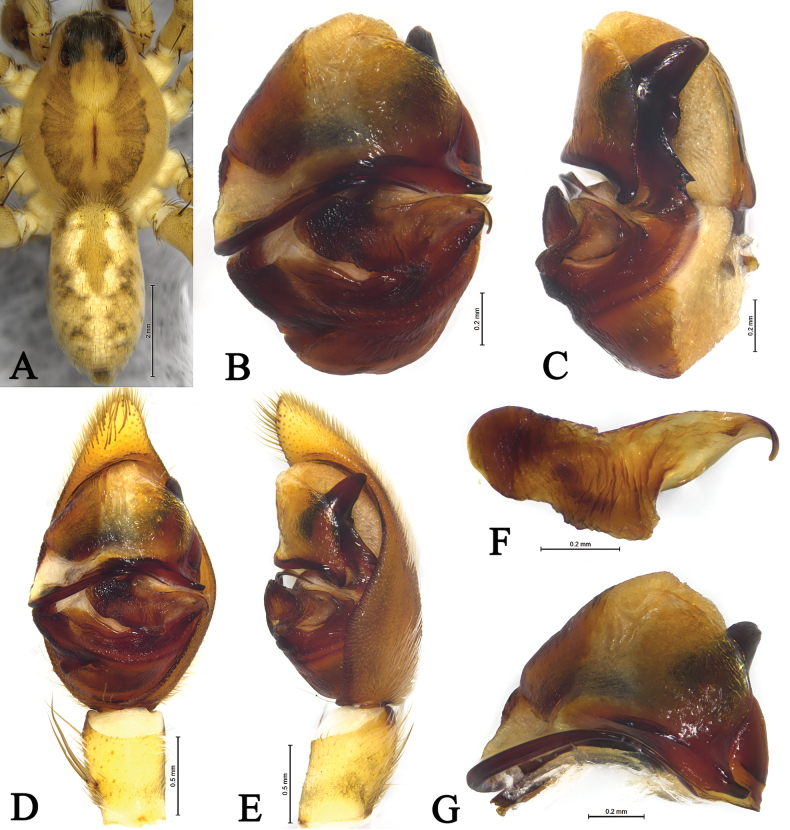
*Draposazhanjiangensis* (Yin, Wang, Peng & Xie, 1995) male from Pitas, Sabah, Malaysia. A. Male habitus, dorsal view; B. Left bulb, ventral view; C. Same, retrolateral view; D. Left male palp, ventral view; E. Same, retrolateral view; F. Median apophysis, ventral view; G. Terminal part in obliquely retrolateral.

**Figure 8.**
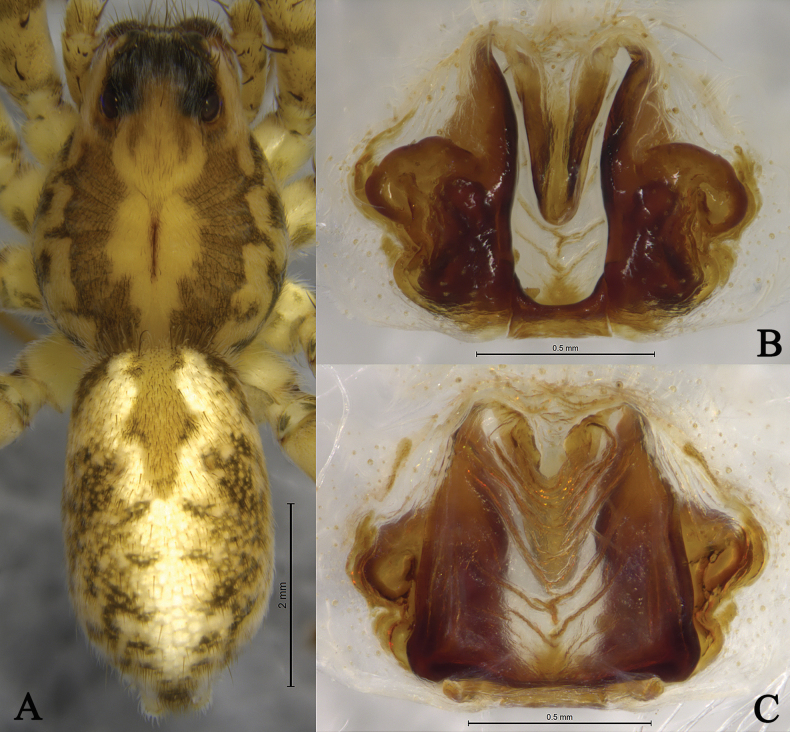
*Draposazhanjiangensis* (Yin, Wang, Peng & Xie, 1995) female from Pitas, Sabah, Malaysia. A. Female habitus, dorsal view; B. Epigyne, ventral view; C. Same, dorsal view.

Figs 1E, F , 5 , 6 , 7 , 8 , 10

Pardosa zhanjiangensis Yin et al., 1995: 74, figs 18–22 (♂♀); Yin et al. 1997b: 281, fig. 133a–e (♂♀); Song et al. 1999: 335, figs 199Q, 200B (♂♀).
Pardosa shugangensis Yin, Bao & Peng, 1997a: 24, figs 39–41 (♂); Yin et al. 1997b: 269, fig. 127a–c (♂); Song et al. 1999: 334, fig. 198M (♂). Syn. nov.

##### Material examined.

**China**: • 1♂ 2♀ (holotype ♀, paratype ♀ and allotype ♂ of *P.zhanjiangensis*), ***Guangdong*** Prov., Zhanjiang City, 25.06.1985, Y.J. Zhang leg. (HNU) • 2♂ (holotype and paratype of *P.shugangensis*), ***Guangxi*** Prov., Beihai City, Shuangang Dadao, 25.11.1995, C.M. Yin leg. (HNU) • **Malaysia: *Sabah***: 6♂ 6♀, Kota Kinabalu, bank of Kawa Kawa R., 06°25'18"N, 116°24'19"E, elev. 50 m, 13.10.2015, L.Y. Wang et al. leg. (SWUC) • 8♂ 8♀, Pitas, 06°41'15"N, 116°57'44"E, elev. 24 m, 20.10.2015, L.Y. Wang et al. leg. (SWUC).

##### Diagnosis.

This species is similar to *D.nicobarica* (Thorell, 1891) (Kronestedt 2010: 44, figs 26, 51–52) and *D.tenasserimensis* (Thorell, 1895) (Kronestedt 2010: 51, figs 28, 54–55) in having strong posterior subpaleal process (PP), hook-like tip of median apophysis, conspicuous deep atrium and tongue-shaped septum, but it can be distinguished by the lamellar, rounded tip in basal half of median apophysis (MA) (vs acute tooth-like), the atrium widens both anteriorly and posteriorly (vs widens posteriorly).

##### Description.

Female holotype of *P.zhanjiangensis* (Fig. 6A) total length 6.69. Carapace 3.34 long, 2.45 wide; opisthosoma 2.66 long, 1.75 wide. Carapace yellow-brown. Cervical groove and radial furrows indistinct. Eye sizes and interdistances: AME 0.15, ALE 0.12, PME 0.40, PLE 0.35; AME–AME 0.10, AME–ALE 0.06, PME–PME 0.29, PME–PLE 0.35. Clypeus height 0.19. Chelicerae yellow-brown. Endites and labium yellowish brown, longer than wide. Sternum yellowish brown, covered with brown setae. Leg measurements: I 9.85 (2.60, 3.57, 2.23, 1.45); II 9.65 (2.70, 3.35, 2.15, 1.45); III 8.76 (2.37, 2.91, 2.22, 1.26); IV 12.63 (3.24, 4.05, 3.75, 1.59). Opisthosoma oval, yellow-brown, and covered with numerous black spots. Heart mark distinct. Venter yellow-brown.

***Epigyne*** (Figs 5C, D, 6C, D, 8B, C). Plate ~1.2 times wider than long. Conspicuous deep atrium (A), atrium widens both anteriorly and posteriorlys; copulatory openings (CO) crack-shaped, as long as atrium. Spermathecal heads (HS) spherical, spermathecal stalks (SS) ~3 times longer than wide, both visible through cuticle of lateral walls of atrium. Fertilization ducts (FD) hook-shaped.

Male allotype of *P.zhanjiangensis* (Fig. 6B) total length 6.62. Carapace 3.56 long, 2.68 wide; opisthosoma 2.92 long, 1.69 wide. Eye sizes and interdistances: AME 0.14, ALE 0.11, PME 0.41, PLE 0.34; AME–AME 0.10, AME–ALE 0.05, PME–PME 0.28, PME–PLE 0.36. Clypeus height 0.22. Leg measurements: I 10.81 (2.74, 3.85, 2.61, 1.61); II 10.19 (2.73, 3.47, 2.47, 1.52); III 9.83 (2.58, 3.31, 2.61, 1.33); IV 14.13 (3.40, 4.48, 4.40, 1.85).

***Palp*** (Figs 5A, B, 6E, F, 7B–G). Tibia yellow-brown, 1.4 times longer than wide. Cymbium yellow-brown, 1.7 times longer than wide. Paleal apophysis triangular (PA). Anterior subpaleal process (AP) strong and rodlike, with a rounded end. Posterior subpaleal process (PP) lamellar, with a notch. Median apophysis (MA) moderately long, with lamellar projection in basal half, subapical protrusion triangular (SAP), tip of median apophysis hook-like. Embolus (E) narrow, evenly tapering to tip.

##### Distribution.

China (Guangxi and Guangdong), Malaysia (Sabah) (Fig. 10). Records from Malaysia (Tioman Island, Sarawak) and Indonesia (Kronestedt 2010) are doubtful and require confirmation.

#### ﻿ Pardosa burasantiensis

Taxon classificationAnimaliaAraneaeLycosidae

Tikader & Malhotra, 1976 sensu Yin et al. (1997b)

711957A2-D0AE-53F7-82F1-F0B8BF8518C1

Figs 9 , 10

Pardosa burasantiensis Tikader & Malhotra, 1976: 130, figs 10–12 (♂♀); Yin et al. 1997b: 239, fig. 112a–g (♂♀, misidentified per Kronestedt 2010: 34); Song et al 1999: 330, figs 194C (♀, misidentified per Kronestedt 2010: 34); Yin et al. 2012: 833, fig. 416a–g (♂♀).
Draposa burasantiensis : Dhali et al., 2012: 1202.
For complete list of references see WSC (2025).

##### Comments.

This species was originally described based on a female holotype and male paratypes collected from Dehradun, Uttar Pradesh, India. Kronestedt (2010) proposed a potential taxonomic reclassification, suggesting that this species might belong to *Draposa*, based on the presence of a subpaleal sclerite in the illustrated male specimen. However, it should be noted that the figure of the paratype male is rather schematic. Morphological examination of the female holotype indicates characteristics consistent with the *nebulosa* species group within *Pardosa* C.L. Koch, 1847. Dhali et al. (2012) transferred two *Pardosa* species (*P.amkhasensis* Tikader & Malhotra, 1976 and *P.burasantiensis* Tikader & Malhotra, 1976) to *Draposa*, referring to Kronestedt (2010) as justification. However, their work lacked substantial morphological evidence to support this reclassification, providing neither detailed descriptions nor clear images of diagnostic characteristics. Study of the paratype female of this species by Dr Souvik Sen from the Zoological Survey of India (Kronestedt pers. comm.) reveals that it belongs to *Pardosa* and male paratype is not conspecific with female. *Pardosaburasantiensis* was first reported from China by Yin et al. (1997b) and figures provided in their publication refer to *Pardosa* and most likely not conspecific with the holotype of *P.burasantiensis*. Therefore, species reported from China does not belong to *Draposa*. In addition, we hereby reinstate the original taxonomic classification for *P.burasantiensis* Tikader & Malhotra, 1976.

**Figure 9.**
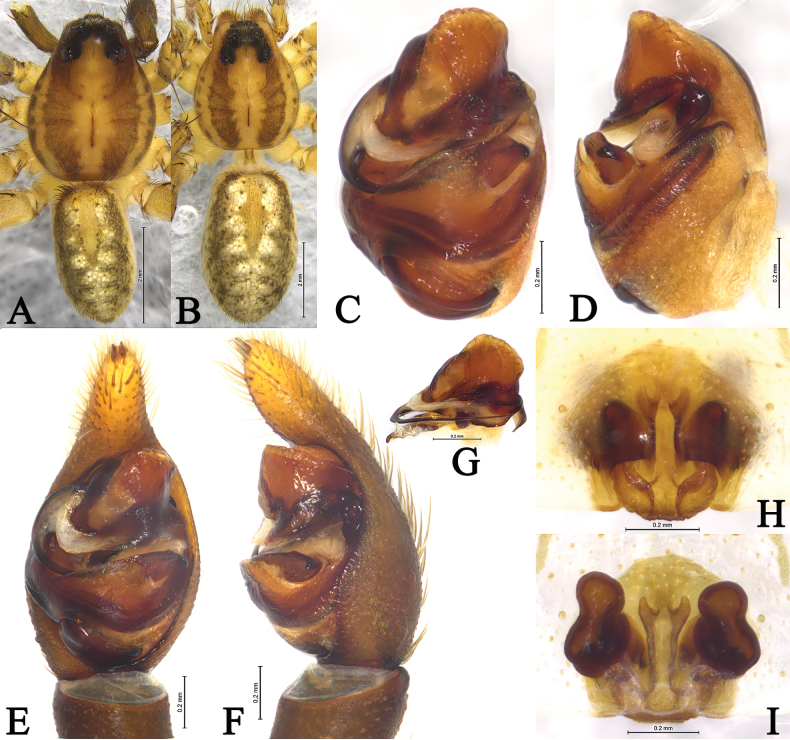
*Pardosaburasantiensis* sensu Yin et al. (1997b) from Puer City, Yunnan, China. A. Male habitus, dorsal view; B. Female habitus, dorsal view; C. Left male palp, bulb, ventral view; D. Same, retrolateral view; E. Left male palp, ventral view; F. Same, retrolateral view; G. Terminal part in obliquely retrolateral; H. Epigyne, ventral view; I. Same, dorsal view.

**Figure 10.**
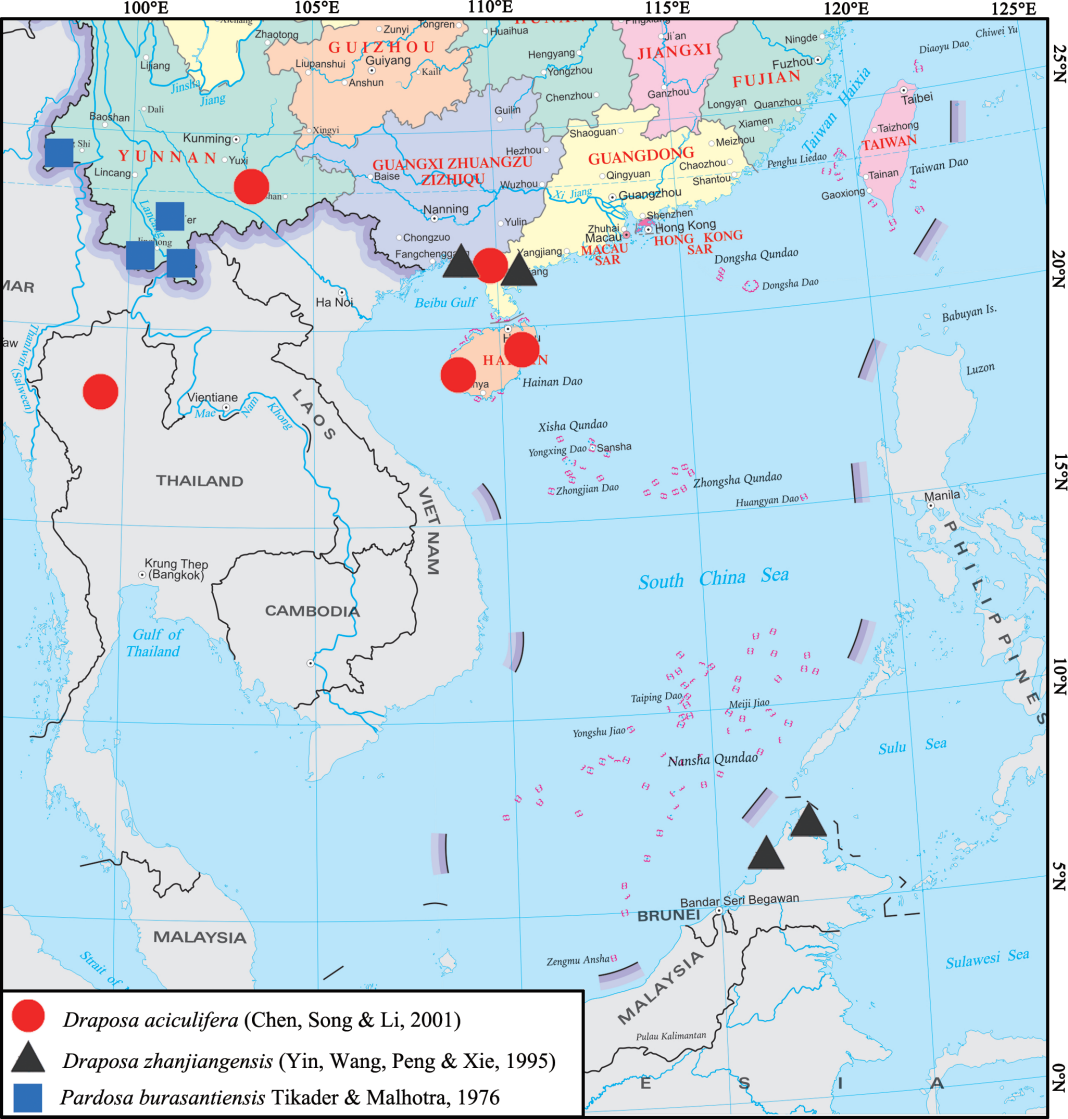
Distribution records of *Draposaaciculifera* (Chen, Song & Li, 2001) and *Draposazhanjiangensis* (Yin, Wang, Peng & Xie, 1995) and Chinese records of *Pardosaburasantiensis* Tikader & Malhotra, 1976.

## Supplementary Material

XML Treatment for Draposa

XML Treatment for Draposa aciculifera

XML Treatment for Draposa zhanjiangensis

XML Treatment for Pardosa burasantiensis

## ﻿Additional information

### Use of AI

No use of AI was reported.

### Author ORCIDs

Zhi-Sheng Zhang https://orcid.org/0000-0002-9304-1789

Piao Liu https://orcid.org/0000-0002-2060-0826

Shuqiang Li https://orcid.org/0000-0002-3290-5416

Lu-Yu Wang https://orcid.org/0000-0002-5250-3473

### Data availability

All of the data that support the findings of this study are available in the main text.
